# Baseline characteristics of patients with acute ischaemic stroke included in the randomised controlled Find-AF 2 trial

**DOI:** 10.1186/s42466-025-00399-8

**Published:** 2025-06-26

**Authors:** Katrin Wasser, Tobias Uhe, Wolf-Rüdiger Schäbitz, Martin Köhrmann, Martin Dichgans, Johannes Brachmann, Ulrich Laufs, Götz Gelbrich, David Petroff, Christiane Prettin, Dominik Michalski, Johann Pelz, Andrea Kraft, Thorleif Etgen, Hassan Soda, Florian Bethke, Peter D. Schellinger, Katharina Althaus, Gerhard F. Hamann, Martin Grond, Bernd Kallmünzer, Martina Petersen, Lars-Peder Pallesen, Michael Ertl, Philipp Zickler, Sven Poli, Karl Georg Haeusler, Thorsten Steiner, Paul Sparenberg, Pawel Kermer, Anna Kopczak, Lars Kellert, Martin Nückel, Jan Liman, Peter Arthur Ringleb, Meinhard Mende, Marcus Wagner, Deborah Bochert, Marlena Schnieder, Imke Amanzada, Sonja Gröschel, Marianne Hahn, Timo Uphaus, Klaus Gröschel, Rolf Wachter, on behalf of the Find-AF 2 study group, Katrin Wasser, Tobias Uhe, Wolf-Rüdiger Schäbitz, Martin Köhrmann, Martin Dichgans, Dominik Michalski, Johann Pelz, Andrea Kraft, Thorleif Etgen, Hassan Soda, Florian Bethke, Katharina Althaus, Martin Grond, Bernd Kallmünzer, Martina Petersen, Lars-Peder Pallesen, Michael Ertl, Sven Poli, Thorsten Steiner, Paul Sparenberg, Pawel Kermer, Anna Kopczak, Lars Kellert, Martin Nückel, Jan Liman, Deborah Bochert, Marlena Schnieder, Timo Uphaus, Klaus Gröschel, Rolf Wachter, Katja Wartenberg, Charlotte Huber, Alhuda Dabbagh, Matthias Kaste, Ahmed Bouchair, Ntalia Meleshchenco, Sevinj Guluzade, Christian Oelschläger, Alkisti Kitsiou, Julian Gehrmeyr, Nikolaos Pantos, Rebecca Seifert, Matthias Korf, Anne Beyer, Christian Mahnkopf, Steffen Schnupp, Sonia Busch, Thomas Mischke, Nawar Alachkar, Marianna Hahn, Arda Civelek, Khadija Mammadli, Benedikt Frank, Benjamin Stolte, Jordi Kühne Escolá, Stefan Kääb, Konstantinos Dimitriadis, Bettina Küster, Moritz Sinner, Maria Kaffe, Philip Melton, Janina Kuffer, Jörg Berrouschot, Anett Stoll, Janina Keilitz, Wiebke Keller, Darius Nabavi, Jens Offermann, Olaf Crome, Boris Dimitrijeski, Carsten Meincke, Gabor Petzold, Felix Jürgen Bode, Sebastian Stösser, Julius Meißner, Taraneh Ebrahimi, Julia Nordsiek, Niklas Beckonert, Christian Thielscher, Omid Shirvani, Andreas Kastrup, Andreas Schröter, Jan Philip Jürß, Volker Pütz, Martin Arndt, Timo Siepmann, Mirko Brudzinski, Frank R. Heinzel, Jennifer Frehe, Kosmala Macha, Matthias Krämer, Alexander Sektita, Niklas Keller, Jan Hedrik Schäfer, Daniel Charisse, Konstantin Kohlhase, Ferdinand Bohmann, Franziska Mayser, Johanna Judith Herget, Franziska Lieschke, Luca Hladek, Tobias Neumann-Haefelin, Jörg Berthel, Lirim Alijaj, Anne Kandler, Burkhard Alber, Robert Müller, Lars Marquardt, Haiko Kazarians, Karin Weissenborn, Hans Worthmann, Ann-Katrin Hennemann, Johanna Ernst, Svenja Jochmann, Jana Al-Ayoubi, Johannes Teller, Ramona Schuppner, Peter Ringleb, Jan Purrucker, Ioana Huber, Simon Schieber, Damjan Mirkov, Carsten Hobohm, Katrin Pomrehn, Peter Schellinger, Jörg Glahn, Jan Schubert, Simone Jenniges, Jens Minnerup, Renate Weinhardt, Erich Hiermann, Jan Birringer, Sandra Lichte-Schneider, Daniela Hütwohl, Rainer Kollmar, Ahmad Ajaz Ganai, Jan Cierpinski, Götz Thomalla, Robert Schulz, Focko Higgen, Felix Nägele, Joachim Röther, Peter Michels, Zoran Vukovic, Robert Berger, Jan Philip Buschmann, Anna Alegiani, Peter Cidlinsky, Peter Nordbeck, Dominik Lehrieder, Thomas Fischer, Christian Hametner, Moritz Huttelmaier, Simona Weiner, Christine Vogl, Stefanie Röckel, Philipp Patrick Zickler, Lino Braadt, Michael Rosenkranz, Stefan Boskamp, Lena Selbel, Christian Schöps, Milena Haase, Alexandra Gómez Expósito, Joshua Mbro, Julia Hummel, Elena Buß, Konstantin Kirchmeier, Liubov Novikova, Lars Udo Krause, Lars Bobowski, Hassan Abou Eid, Esteban Vajda-Medina, Mareike Probst, Hisham Essa, Bernhard Eberl, Dragana Milankovic-Eberl, Georg Rieder, Olav Schwarte, Constanze Höcherl, Frank Hoffmann, Bettina Schönmuth, Anja Giebler, Rafaela Voss, Katrin Sachadae, Oliver Bähr, Herbert Gruber, Sonka Benesch, Thomas Pollinger, Avinash Suntha, Jana Fett, Katja Burian, Mirko Seidel, Daniel Peters, Stephan Lenze, Judith Becher, Katja Bahcesular, Florans Pfeiffer, Sophia Potthoff, Mario Enrique de la Piedra Walter, Bettina Schmitz, Carolin Waldschmidt, Anna Gutwinski, Veronika Angermüller, Tameem Alhammoud, Hüsniye Cakiroglu, Jörg Müller, Martin Honermann, Marian Christoph Burgstaller, Herwig Strik, Arne Lenz, Mark Obermann, Nicoletta Adochitei, Frank Arne Wollenweber, Laya Rahban, Matthias Julius Grosch, Erik Ellwardt, Martin Jünemann, Omar AlHaj Omar, Christian Fräbel, Toska Maxhuni, Christian Claudi, Oliver Posner, Francesca Culay, Waltraud Pfeilschifter, Christoffer Kraemer, Milena Wiemers, Micha Simon, Alexander Finke, Ondrej Hlavac

**Affiliations:** 1 Department of Neurology, University of Göttingen Medical Centre, Göttingen, Germany; 2 Department of Cardiology, University of Leipzig Medical Center, Liebigstraße 20, Haus 4, 04103 Leipzig, Germany; 3 Department of Neurology, Evangelisches Klinikum Bethel, University Hospital OWL of the University Bielefeld, Campus Bielefeld-Bethel, Bielefeld, Germany; 4 Department of Neurology and Centre for Translational Neuro- and Behavioral Sciences (C-TNBS), University Hospital Essen, Essen, Germany; 5 Institute for Stroke and Dementia Research (ISD), University Hospital, LMU Munich, Munich, Germany; 6 German Centre for Neurodegenerative Diseases (DZNE, Munich), Munich, Germany; 7 Munich Cluster for Systems Neurology (SyNergy), Munich, Germany; 8 Department of Cardiology, Klinikum Coburg, Coburg, Germany; 9 Institute for Clinical Epidemiology and Biometry, University of Würzburg, Würzburg, Germany; 10 Clinical Trial Centre Würzburg, University Hospital Würzburg, Würzburg, Germany; 11 Clinical Trial Centre (ZKS) Leipzig, Leipzig University, Leipzig, Germany; 12 Department of Neurology, University of Leipzig Medical Center, Leipzig, Germany; 13 Department of Neurology, Hospital Martha-Maria, Halle, Germany; 14 Department of Neurology, Klinikum Traunstein, Traunstein, Germany; 15 Department of Neurology, Rhön Hospital, Bad Neustadt, Germany; 16 Department of Neurology, Ibbenbüren Hospital, Ibbenbüren, Germany; 17 Department of Neurology and Neurogeriatrics, University Hospital Minden, UK RUB, Minden, Germany; 18 Department of Neurology, University Hospital Ulm, Ulm, Germany; 19 Department of Neurology and Neurological Rehabilitation, Bezirkskrankenhaus Günzburg, Günzburg, Germany; 20 Department of Neurology, Siegen Hospital, Siegen, Germany; 21 Department of Neurology, Friedrich-Alexander-Universität Erlangen-Nürnberg (FAU), Erlangen, Germany; 22 Department of Neurology, Klinikum Osnabrück, Osnabrück, Germany; 23 Centre of Clinical Neuroscience, Department of Neurology, Medical Faculty and University Hospital Carl Gustav Carus, TU Dresden, Dresden, Germany; 24 Clinic for Neurology and Clinical Neurophysiology, Faculty of Medicine, University of Augsburg, Augsburg, Germany; 25 Department of Vascular Neurology, Eberhard-Karls University, Tuebingen, Germany; 26 Department of Neurology, Universitätsklinikum Würzburg (UKW), Würzburg, Germany; 27 Department of Neurology, Klinikum Frankfurt Höchst, Frankfurt, Germany; 28 Clinic for Neurology, BG Klinikum Unfallkrankenhaus Berlin, Berlin, Germany; 29 Department of Neurology, Friesland Kliniken, Sande, Germany; 30 Department of Neurology, University Hospital, LMU Munich, Munich, Germany; 31 Clinic for Neurology, Klinikum Nürnberg, Nuremberg, Germany; 32 Department of Neurology, Ruprecht Karls University Heidelberg, Heidelberg, Germany; 33 Institute for Medical Informatics, Statistics and Epidemiology (IMISE), Leipzig University, Leipzig, Germany; 34 Department of Neurology, University Medical Centre of the Johannes Gutenberg University Mainz, Mainz, Germany; 35 DZHK (German Centre for Cardiovascular Research), Partner Site, Göttingen, Germany; 36 Department of Cardiology, University of Göttingen Medical Centre, Göttingen, Germany

**Keywords:** Stroke, Atrial fibrillation, ECG monitoring, Randomised trial

## Abstract

**Background:**

In the Find-AF 2 randomised controlled trial, we investigate whether a risk-adapted intensified heart rhythm monitoring with subsequent initiation of oral anticoagulation in ischaemic stroke patients leads to a reduction of recurrent ischaemic stroke and systemic embolism. The objective of this analysis is to present baseline characteristics of the overall Find-AF 2 study population and stratified by low or high risk for developing AF.

**Methods:**

The Find-AF 2 trial included acute ischaemic stroke patients ≥ 60 years of age within 30 days of ischaemic stroke of any cause. Before randomisation, patients received a 24-h Holter-ECG to exclude those with easily detectable AF and to determine the presence or absence of enhanced supraventricular ectopic activity (ESVEA), used as a marker indicating high or low risk for developing AF. Those without AF were randomly assigned 1:1 to either usual care diagnostics for AF detection (control group) or enhanced, prolonged and intensified ECG monitoring (intervention group). In the intervention group, patients with ESVEA received an implantable cardiac monitor (ICM), whereas those without ESVEA received repeated annual 7-day Holter ECGs. We present baseline characteristics of the overall Find-AF 2 population and stratified by ESVEA.

**Results:**

Between July 2020 and July 2024, 5227 patients (mean age 72.3 ± 7.5 years, 40% female, 2618 intervention group, 2609 control group) were randomised from 52 study centres in Germany within a median of 5 (IQR 3–7) days after the index stroke. The most frequent stroke aetiologies were cryptogenic (60%) and small vessel occlusion (19%). 1152 (22%) patients were at high risk for developing AF and 4075 (78%) at low risk. Patients within the high-risk stratum were significantly older (mean age 75.2 versus 71.5 years, *p* < 0.001), more often had moderate to severe stroke (34% versus 30%, *p* < 0.001), non-lacunar (70% versus 64%, *p* < 0.001) and of cryptogenic aetiology (64% vs 58%, *p* < 0.001).

**Conclusions:**

The Find-AF 2 trial has successfully completed recruitment of a large acute ischaemic stroke population with different stroke subtypes. The follow-up is ongoing and results are expected within two years.

**Trial registration:**

ClinicalTrials.gov, Identifier NCT04371055, registered 24 April 2020.

**Supplementary Information:**

The online version contains supplementary material available at 10.1186/s42466-025-00399-8.

## Background

Atrial fibrillation (AF) accounts for up to 20% of ischaemic strokes [1, 2], but is probably underestimated because detection of paroxysmal AF during routine diagnostics is rather challenging. The detection of previously undetected AF is clinically important since the risk of recurrent stroke in patients with clinical AF can be reduced by oral anticoagulation (OAC) [3]. Six randomised controlled trials and a subsequent meta-analysis have proved that prolonged heart rhythm monitoring after ischaemic stroke improves AF detection and leads to greater use of OAC [4–10]. However, whether prolonged heart rhythm monitoring also leads to lower rates of recurrent embolic events need to be elucidated [11]. Furthermore, limited resources may necessitate identification of sub-populations at particularly high risk of having or developing AF.

Various concepts for risk stratification of ischaemic stroke patients who could benefit from OAC have been proposed. One approach is to draw conclusions on the risk of AF from the stroke types according to the Trial of ORG 10172 in Acute Stroke Treatment (TOAST) classification [12]. Alternatively, stroke patients potentially benefitting from OAC have been identified based on the embolic stroke of unknown source (ESUS) concept [13]. The underlying hypothesis of this concept was that most cryptogenic strokes in patients with a suitable lesion distribution pattern on computed tomography (CT) or magnetic resonance imaging (MRI) are due to embolic events and recurrent strokes might therefore be prevented by OAC. Several randomised trials comparing different direct oral anticoagulants with acetylsalicylic acid failed to show a general superiority of direct OACs compared to aspirin in the overall ESUS population [14, 15]. Subsequently, “enriched ESUS concepts” were examined with the addition of clinical, echocardiographic and ECG parameters, non-stenotic plaques or laboratory parameters such as natriuretic peptides [16–18]. A common feature of all these conventional or modified ESUS concepts is that they demonstrate a reduction in recurrent strokes only in subgroup analyses, but failed to show a significant benefit in the overall study populations.

In the Find-AF 2 trial, we assigned risk categories for developing AF based on a standardized 24-h heart rhythm monitoring before randomisation and have chosen the intensity of prolonged heart rhythm monitoring accordingly. As a criterion suggesting a high risk for AF, excessive supraventricular ectopic activity (ESVEA) was used, which has been shown to be associated with the prevalence of AF, stroke and all-cause mortality in population-based cohort of the Copenhagen Holter Study [19]. This was verified in a meta-analysis encompassing 20 trials and over 23,000 participants [20]. Especially in ischaemic stroke patients, ESVEA has been shown to identify patients with an increased risk of paroxysmal AF [21–23] as well as an increased risk of stroke recurrence [19, 24].

In the Find-AF 2 trial all patients from the intervention arm received prolonged heart rhythm monitoring. Those patients from the intervention group with ESVEA received continuous ECG monitoring by ICM (intensified monitoring), whereas patients without ESVEA received repeated 7-day Holter ECGs. All ECGs were evaluated in a specialised central ECG core lab (enhanced monitoring).

Here, we provide a report on key baseline characteristics of the entire Find-AF 2 cohort according to the randomisation arms and the risk categories for developing AF. Understanding the at-risk population for AF might be important for a preselection of ischaemic stroke patients in which enhanced, prolonged and intensified heart rhythm monitoring for AF could be particularly effective.

## Methods

### Study design

The ongoing Find-AF 2 trial (NCT04371055) is a randomised, controlled prospective open-label multicentre trial with blinded endpoint assessment (PROBE) conducted at 52 study centres with board certified stroke units in Germany. The study rationale and design have been published previously [25]. Briefly summarized, Find-AF 2 investigates whether, in patients with acute ischaemic stroke, a risk-adapted approach of enhanced, prolonged and intensified ECG monitoring to detect underlying AF results in a reduction of recurrent ischaemic stroke and systemic embolism compared to standard ECG monitoring practices consisting of at least 24-h ECG monitoring by tailoring anticoagulation to those with newly detected AF. The primary endpoint is the time to first recurrent ischaemic stroke or systemic embolism. The study protocol has been approved by local ethics boards at each study site.

### Participants

Eligible patients were ≥ 60 years of age and suffered an acute ischaemic stroke within the last 30 days. An ischaemic stroke was defined as a sudden focal neurologic deficit lasting at least 24 h with matching signs of stroke within the territory of a major cerebral artery. If neurologic deficits lasted less than 24 h, a corresponding lesion on brain imaging such as an acute lesion on diffusion-weighted MR imaging, non-contrast CT or CT perfusion imaging or an occlusion or intravascular thrombus on CT, magnetic resonance or digital subtraction angiography was mandatory as inclusion criterion. Patients with and without a typical embolic infarction pattern could be included. Main exclusion criteria were known AF, given indication or contraindication for OAC, history of intracranial bleeding or a carotid artery stenosis needing operation or intervention within the last or upcoming 30 days. All other ischaemic stroke aetiologies were eligible.

### Study procedures

At screening, all study participants received a 24-h Holter ECG. If AF was found, then these patients were excluded from further study participation. Patients with either ≥ 30 premature atrial contractions/hour × recorded hours or at least one atrial run ≥ 20 beats fulfilled the ESVEA criteria and were assigned to the high-risk stratum. After evaluating the baseline 24-h Holter ECGs in a specialised central ECG core lab, all eligible patients without AF were randomised 1:1 to intervention or usual care. Patients in the intervention group within the high-risk stratum were offered continuous heart rhythm monitoring by ICM. Only Reveal Linq® event recorders from Medtronic were used. Those in the intervention group in the low-risk stratum received 7-day Holter ECGs after randomisation, 3 months and annually thereafter. In the control arm, no information regarding the stratum was disclosed to patients or study investigators. All study ECGs were analysed in a central ECG core-laboratory, blinded to patient characteristics. Usual care procedures comprised stroke unit telemetry and additional Holter ECGs at the discretion of the treating physicians.

### Statistical analysis

Descriptive statistics comprise means ± standard deviation or median [interquartile range] for continuous variables and frequencies for categorical variables. The *p*-values for comparisons between groups were derived from (generalized) linear models with sex and age as covariates, except when age and sex were the dependent variables. All analyses were performed with the software R version 4.4.0. [26].

## Results

### Study conduct and participants

**Fig. 1:**
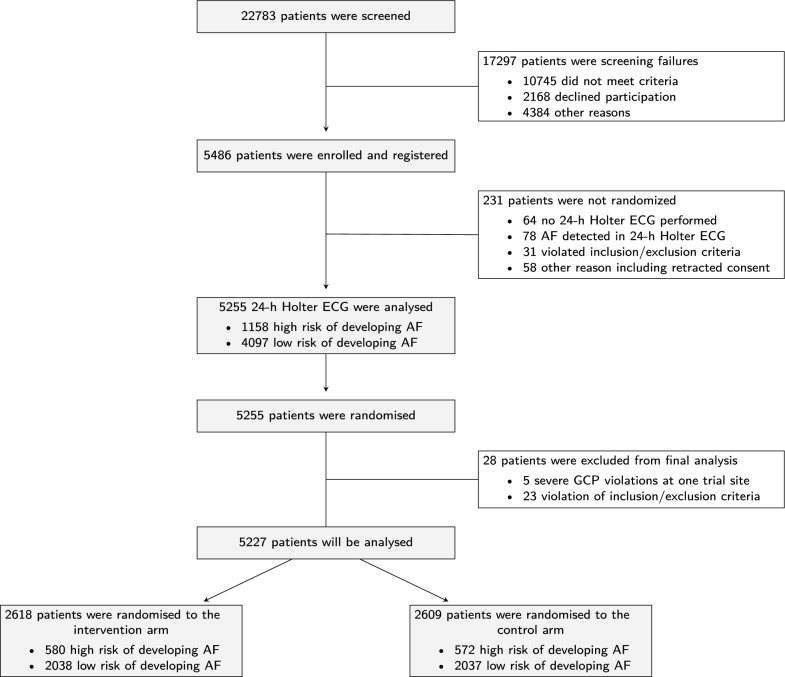
CONSORT participant flow diagram at recruitment

A total of 5486 ischaemic stroke patients ≥ 60 years initially fulfilled all inclusion and exclusion criteria and were registered and enrolled in Find-AF 2 in 52 study centres in Germany between July 6th, 2020 and July 1st, 2024 (Fig. 1). After exclusion of 231 participants before randomisation (most often due to detection of AF within 24-h Holter ECG) and exclusion of 28 participants after randomisation (most often due to violation of inclusion/exclusion criteria detected very shortly after randomisation), 5227 patients were eligible for final analysis of whom 2618 (50.1%) were randomised to the intervention and 2609 (49.9%) to the control group. In both study groups, 22% of the patients fulfilled the chosen ESVEA criteria in the 24-h Holter ECG and therefore, were anticipated to be at high risk of developing AF (580 in the intervention group and 572 in the control group).

### Baseline characteristics of the overall study population

Demographics and characteristics of the entire study population are summarised in Table 1. For the few missing baseline data we refer to Table S1 for details.

**Table 1:** Baseline characteristics of the entire Find-AF2 study population

	All patients (n = 5227)	Control (n = 2609)	Intervention (n = 2618)
Number of females	2115 (40%)	1039 (40%)	1076 (41%)
Age (years)	72.3 ± 7.5	72.3 ± 7.5	72.3 ± 7.6
< 70	2230 (43%)	1109 (43%)	1121 (43%)
70 to < 75	1112 (21%)	556 (21%)	556 (21%)
≥ 75	1885 (36%)	944 (36%)	941 (36%)
BMI (kg/m^2^)	27.4 ± 4.7	27.5 ± 4.7	27.4 ± 4.7
Smoking			
Never	2680 (53%)	1350 (53%)	1330 (52%)
Ex-smoker	1573 (31%)	769 (30%)	804 (32%)
Current smoker	839 (16%)	424 (17%)	415 (16%)
Alcohol consumption			
Abstinent	2515 (50%)	1251 (50%)	1264 (50%)
Moderate	2219 (44%)	1127 (45%)	1092 (44%)
Heavy	290 (6%)	139 (6%)	151 (6%)
Medical history			
Previous stroke	716 (14%)	343 (13%)	373 (14%)
Previous TIA	216 (4%)	103 (4%)	113 (4%)
Systemic embolism	58 (1%)	28 (1%)	30 (1%)
Heavy bleeding	39 (1%)	17 (1%)	22 (1%)
Coronary artery disease	567 (11%)	283 (11%)	284 (11%)
Myocardial infarction	318 (6%)	149 (6%)	169 (6%)
Heart failure	201 (4%)	106 (4%)	95 (4%)
Peripheral artery disease	137 (3%)	69 (3%)	68 (3%)
Currently requires dialysis	8 (0%)	2 (0%)	6 (0%)
Diabetes mellitus	1298 (25%)	628 (24%)	670 (26%)
Arterial hypertension	4127 (79%)	2061 (79%)	2066 (79%)
Dyslipidaemia	2710 (52%)	1325 (51%)	1385 (53%)
High risk of AF	1152 (22%)	572 (22%)	580 (22%)
Time from index stroke to randomisation	5.0 [3.0, 7.0]	5.0 [3.0, 7.0]	5.0 [4.0, 7.0]
≤ 3 days	1327 (25%)	693 (27%)	634 (24%)
4–7 days	2678 (51%)	1314 (50%)	1364 (52%)
8–14 days	906 (17%)	439 (17%)	467 (18%)
> 14 days	315 (6%)	163 (6%)	152 (6%)
Index stroke			
Lacunar	1158 (22%)	572 (22%)	586 (22%)
Non-lacunar	3428 (66%)	1713 (66%)	1715 (66%)
Not identifiable/unknown	641 (12%)	324 (12%)	317 (12%)
TOAST classification of index stroke			
Large-artery atherosclerosis (LAA)	461 (9%)	228 (9%)	233 (9%)
Cardioembolism (CE)	543 (10%)	284 (11%)	259 (10%)
Small vessel occlusion (SVO)	990 (19%)	495 (19%)	495 (19%)
Other cause (OC)	96 (2%)	49 (2%)	47 (2%)
Unknown cause (UC)	3099 (60%)	1536 (59%)	1563 (60%)
ESUS			
Yes	2655 (51%)	1301 (50%)	1354 (52%)
No	2078 (40%)	1050 (40%)	1028 (39%)
Unknown/missing	494 (9%)	258 (10%)	236 (9%)
I.v. or i.a. thrombolysis	1361 (26%)	714 (27%)	647 (25%)
NIH Stroke Scale at admission	2.0 [1.0, 4.0]	2.0 [1.0, 4.0]	2.0 [1.0, 4.0]
Minor stroke (1–3)	3633 (70%)	1791 (69%)	1842 (70%)
Moderate stroke (4–10)	1375 (26%)	715 (27%)	660 (25%)
Severe stroke (11–43)	219 (4%)	103 (4%)	116 (4%)
CHADS_2_ score prior to stroke	1.8 ± 1.2	1.8 ± 1.2	1.8 ± 1.2
0	657 (13%)	329 (13%)	328 (13%)
1	1749 (33%)	897 (34%)	852 (33%)
2	1607 (31%)	803 (31%)	804 (31%)
3	692 (13%)	320 (12%)	372 (14%)
4	397 (8%)	200 (8%)	197 (8%)
5	111 (2%)	52 (2%)	59 (2%)
6	10 (0%)	8 (0%)	2 (0%)
CHA_2_DS_2_-VA score prior to stroke	2.6 ± 1.4	2.6 ± 1.4	2.7 ± 1.4

Numbers are counts (percentage), mean ± standard deviation or median [interquartile range]. Regarding alcohol, abstinent means zero dpw, and heavy is more than 14 dpw (m) or 7 dpw (f) based on the National Institute on Alcohol Abuse and Alcoholism (NIAAA). We termed less alcohol intake to be ‘moderate’. The term ‘Unknown’ means that the CRF has been filled out with this term whereas ‘Missing’ means that the item has not been filled out. dpw = drinks per week

CHADS_2_: Congestive heart failure, arterial hypertension, diabetes mellitus, history of stroke/transient ischaemic attack (doubled). CHA_2_DS_2_-VA: congestive heart failure, arterial hypertension, age ≥75 years (doubled), diabetes mellitus, history of stroke/transient ischaemic attack (doubled), vascular disease, age 65 to 74 years [40]

As expected in a randomised trial, baseline characteristics were well matched between the intervention and the control group.

The mean age was 72.3 ± 7.5 years and 40% were female. The most frequent cardiovascular risk factors were arterial hypertension (79%), dyslipidaemia (52%) and diabetes mellitus (25%). The mean CHADS_2_ (congestive heart failure, arterial hypertension, age, diabetes mellitus, previous stroke/transient ischaemic attack (TIA)) score was 3.4 ± 0.9. 14% had previous stroke and 4% previous TIA, before the index stroke.

Although the inclusion criteria allowed for index strokes up to 30 days prior to enrolment, Table 1 shows that over 75% of patients were included within 7 days after the index stroke. The National Institutes of Health Stroke Scale (NIHSS) score at admission was mostly low. Only 4% suffered from severe (NIHSS > 10 points) and 26% from moderate strokes (NIHSS 4–10 points) [27], i.e. 70% had minor strokes (NIHSS ≤ 3 points) [28]. With regard to stroke aetiology according to the TOAST classification at hospital discharge, 3099 patients in the trial had a cryptogenic stroke (60%). Of them, 470 (15%) had ≥ 2 causes, for 2313 (75%) no cause was discernible and for 262 (8%) the evaluation was incomplete. There were 54 patients with cryptogenic stroke for whom no further data were provided. The second most common cause of stroke according to the TOAST classification was small vessel occlusion stroke (19%).

### Baseline characteristics by high or low risk stratum

In 1152 patients (22%), the initial 24-h Holter ECG showed ESVEA meaning that these patients met the chosen criterion suggesting high risk for developing AF. The remaining 4077 patients (78%) did not fulfil the criteria of ESVEA and were therefore considered to be at low risk for developing AF.

An overview of the baseline characteristics of the study population stratified by high and low risk of developing AF is displayed in Table 2. Briefly, patients in the high-risk stratum were significantly older (75.2 ± 7.5 versus 71.5 ± 7.3 years, *p* < 0.001), whereas no significant difference in sex distribution was observed. Index stroke types also differed significantly (*p* < 0.001): Non-lacunar strokes occurred more frequently (70% versus 64%) in patients at high risk for AF. Index stroke aetiology was assessed at discharge. No reclassification of stroke aetiology was done during follow-up. The distribution of index stroke aetiology according to TOAST showed significant differences (*p* < 0.001): Small vessel occlusion strokes occurred less frequently in the high-risk population (14% versus 20%), whereas the rate of cryptogenic stokes was higher (64% versus 58%). In addition, index strokes were slightly more severe in patients in the high-risk stratum according to the admission NIHSS (*p* < 0.001).

**Table 2:** Baseline characteristics by low/high-risk of AF based on excessive supraventricular ectopic activity (ESVEA) within 24-h Holter-ECG

	All patients (n = 5227)	Low risk for AF (n = 4075)	High risk for AF (n = 1152)	*P*-value
Number of females	2115 (40%)	1657 (41%)	458 (40%)	0.58
Age (years)	72.3 ± 7.5	71.5 ± 7.3	75.2 ± 7.5	** < 0.001**
< 70	2230 (43%)	1924 (47%)	306 (27%)	
70 to < 75	1112 (21%)	859 (21%)	253 (22%)	
≥ 75	1885 (36%)	1292 (32%)	593 (51%)	
BMI (kg/m^2^)	27.4 ± 4.7	27.5 ± 4.7	27.4 ± 4.9	**0.047**
Smoking				** < 0.001**
Never	2680 (53%)	2025 (51%)	655 (59%)	
Ex-smoker	1573 (31%)	1244 (31%)	329 (30%)	
Current smoker	839 (16%)	710 (18%)	129 (12%)	
Alcohol consumption				0.33
Abstinent	2515 (50%)	1964 (50%)	551 (50%)	
Moderate	2219 (44%)	1739 (44%)	480 (43%)	
Heavy	290 (6%)	216 (6%)	74 (7%)	
Medical history				
Previous stroke	716 (14%)	565 (14%)	151 (13%)	0.094
Previous TIA	216 (4%)	159 (4%)	57 (5%)	0.50
Systemic embolism	58 (1%)	42 (1%)	16 (1%)	0.43
Heavy bleeding	39 (1%)	27 (1%)	12 (1%)	0.23
Coronary artery disease	567 (11%)	432 (11%)	135 (12%)	0.48
Myocardial infarction	318 (6%)	242 (6%)	76 (7%)	0.96
Heart failure	201 (4%)	143 (4%)	58 (5%)	0.32
Peripheral artery disease	137 (3%)	104 (3%)	33 (3%)	0.64
Currently requires dialysis	8 (0%)	7 (0%)	1 (0%)	0.52
Diabetes mellitus	1298 (25%)	1013 (25%)	285 (25%)	0.48
Arterial hypertension	4127 (79%)	3178 (78%)	949 (82%)	0.21
Dyslipidaemia	2710 (52%)	2132 (53%)	578 (50%)	0.16
Time from index stroke to randomisation	5.0 [3.0, 7.0]	5.0 [3.0, 7.0]	5.0 [4.0, 7.0]	0.51
≤ 3 days	1327 (25%)	1044 (26%)	283 (25%)	
4–7 days	2678 (51%)	2077 (51%)	601 (52%)	
8–14 days	906 (17%)	714 (18%)	192 (17%)	
> 14 days	315 (6%)	239 (6%)	76 (7%)	
Index stroke				** < 0.001**
Lacunar	1158 (22%)	936 (23%)	222 (19%)	
Non-lacunar	3428 (66%)	2620 (64%)	808 (70%)	
Not identifiable/unknown	641 (12%)	519 (13%)	122 (11%)	
TOAST classification of index stroke				** < 0.001**
Large-artery atherosclerosis (LAA)	461 (9%)	359 (9%)	102 (9%)	
Cardioembolism (CE)	543 (10%)	416 (10%)	127 (11%)	
Small vessel occlusion (SVO)	990 (19%)	825 (20%)	165 (14%)	
Other cause (OC)	96 (2%)	78 (2%)	18 (2%)	
Unknown cause (UC)	3099 (60%)	2363 (58%)	736 (64%)	
ESUS				0.078
Yes	2655 (51%)	2042 (51%)	613 (54%)	
No	2078 (40%)	1651 (41%)	427 (37%)	
Unknown/missing	430 (8%)	328 (8%)	102 (9%)	
I.v. or i.a. thrombolysis	1361 (26%)	1041 (26%)	320 (28%)	0.076
NIH Stroke Scale at admission				** < 0.001**
Minor stroke (1–3)	3633 (70%)	2870 (70%)	763 (66%)	
Moderate stroke (4–10)	1375 (26%)	1059 (26%)	316 (27%)	
Severe stroke (11–43)	219 (4%)	146 (4%)	73 (6%)	
CHADS_2_ score prior to stroke	1.8 ± 1.2	1.7 ± 1.2	2.0 ± 1.2	0.74
0	657 (13%)	572 (14%)	85 (7%)	
1	1749 (33%)	1420 (35%)	329 (29%)	
2	1607 (31%)	1171 (29%)	436 (38%)	
3	692 (13%)	529 (13%)	163 (14%)	
4	397 (8%)	287 (7%)	110 (10%)	
5	111 (2%)	84 (2%)	27 (2%)	
6	10 (0%)	8 (0%)	2 (0%)	
CHA_2_DS_2_-VA score prior to stroke	2.6 ± 1.4	2.6 ± 1.4	3.0 ± 1.3	0.94

Numbers are counts (percentage), mean ± standard deviation or median [interquartile range]. Regarding alcohol, abstinent means zero dpw, and heavy is more than 14 dpw (m) or 7 dpw (f) based on the National Institute on Alcohol Abuse and Alcoholism (NIAAA). We termed less alcohol intake to be ‘moderate’. The term ‘Unknown’ means that the CRF has been filled out with this term whereas ‘Missing’ means that the item has not been filled out. dpw = drinks per week. P values below 0.05 are considered significant and shown in bold

### Comparison of the Find-AF 2 trial population with previous ischaemic stroke trial populations

**Fig. 2:**
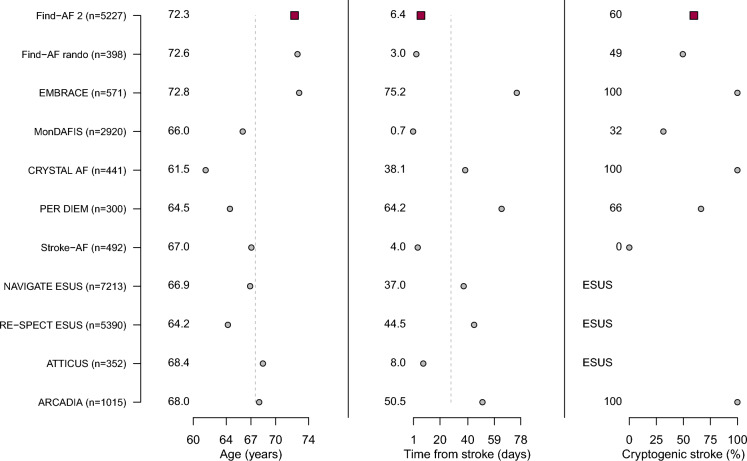
Baseline characteristics in comparison to other ischaemic stroke populations. See Fig. 1 for a comparison between baseline data in Find-AF 2 and other ischaemic stroke trials. The trials depicted are Find-AF 2 [25], Find-AF_rando_ [6], EMBRACE [4], MonDAFIS [9], CRYSTAL AF [5], PER DIEM [7], Stroke-AF [8], NAVIGATE ESUS [14], RE-SPECT ESUS [15], ATTICUS [16], and ARCADIA [17]. The vertical dashed lines represent the weighted mean over all the trials (weights according to patient numbers)

We compared baseline characteristics and heart rhythm monitoring strategies of the Find-AF 2 trial population with previous heart rhythm monitoring trials [4–9] in ischaemic stroke patients as well as with ESUS trial populations [14–17] (see Table 3, Fig. 2).

**Table 3:** Comparison of baseline characteristics between different acute ischemic stroke trials

N	Find-AF 2	Find-AF randomised	EMBRACE	MonDAFIS	CRYSTAL AF	PER DIEM	Stroke-AF	NAVIGATE ESUS	RE-SPECT ESUS	ATTICUS	ARCADIA
	5227	398	571	2920	441	300	492	7213	5390	352	1015
Type and duration of heart rhythm monitoring (intervention vs. control arm)	ICM or repeated 7-day Holter ECG vs. usual care (at least 24-h Holter ECG)	3 × 10-day Holter ECG vs. usual care	30-day external cardiac monitor vs. 24-h Holter ECG	Up to 7-day Holter ECG vs. usual care	ICM vs. usual care	ICM vs. 30-day external cardiac monitor	ICM vs. usual care	n.a.*	n.a.*	n.a.*	n.a.*
Sex (female)	2115 (40%)	160 (40%)	257 (45%)	1139 (39%)	161 (37%)	121 (40%)	185 (38%)	2777 (38%)	1987 (37%)	171 (49%)	551 (54%)
Age (years)	72.3 ± 7.5	72.6 ± 7.4	72.8 ± 8.7	66.0 ± 12.5	61.5 ± 11.4	64.5 ± 12.8	67.1 ± 9.4	66.9 ± 9.8	64.2 ± 11.4	68.5 ± 10.5	68.0 ± 10.6
Diabetes mellitus	1298 (25%)	108 (28%)	110 (19%)	743 (25%)	72 (16%)	60 (20%)	187 (38%)	1806 (25%)	1224 (23%)	100 (28%)	315 (31%)
Arterial hypertension	4125 (79%)	316 (79%)	395 (69%)	2256 (77%)	271 (61%)	187 (62%)	397 (81%)	5580 (77%)	3981 (74%)	303 (86%)	784 (77%)
Dyslipidaemia	2705 (52%)	164 (41%)	368 (64%)	1570 (54%)	253 (57%)			5604 (78%)**	3043 (56%)		
Time from index stroke to randomisation (days)	6.3 ± 4.7	3.0 ± 2.2	75.2 ± 38.6	0.7 ± 0.3***	38.1 ± 27.6	64.2 ± 58.2	4.0 ± 3.0	37.0 ± 54.3	44.5 ± 44.1	8.0 ± 4.4	50.5 ± 48.0
TOAST											
LAA	461 (9%)	7 (2%)	–	803 (28%)	–	26 (9%)	282 (58%)				–
CE	543 (10%)	75 (19%)	–	364 (12%)	–	19 (6%)	–				–
SVO	990 (19%)	118 (30%)	–	756 (26%)	–	48 (16%)	208 (42%)				–
OC	96 (2%)	1 (0%)	–	66 (3%)	–	8 (3%)	–				–
UC	3099 (60%)	197 (49%)	571 (100%)	919 (32%)	441 (100%)	199 (66%)	–	ESUS	ESUS	ESUS	10150%)

*Not applicable in ESUS trials, **In NAVIGATE ESUS dyslipidaemia was defined as “statin use after randomisation”, ***In MonDAFIS the median time from stroke unit admission to randomisation was 16·8 h (10·7–20·8) [9]

TOAST: Trial of ORG 10172 in Acute Stroke Treatment, LAA = large artery atherosclerosis, CE = cardioembolism, SVO = small vessel occlusion, OC = other cause, UC = unknown cause

In comparison to monitoring as well as ESUS studies, patients in Find-AF 2 were slightly older, yet had similar sex ratio and distribution of cardiovascular risk factors. With regard to heart rhythm monitoring studies, differences were found in the duration and type of cardiac rhythm monitoring as well as the time of randomisation after the index stroke (range: 0.7 to 75.2 days, Table 3). A key difference between Find-AF 2 and most other studies was that Find-AF 2 enrolled patients with almost all stroke aetiologies.

## Discussion

The Find-AF 2 trial addresses three major gaps in the management of patients with acute ischaemic strokes:

1. It is the first adequately powered randomised controlled trial with completed recruitment investigating clinical benefits of a standardized strategy of AF screening for secondary stroke prevention.
2. It investigates AF screening strategies irrespective of the suspected stroke aetiology, thereby augmenting previous trials confined to stroke of unknown origin or ESUS [4, 5, 8].
3. It is the first randomised trial investigating an ECG-based risk stratification to adjust the intensity of prolonged heart rhythm monitoring.

### Baseline characteristics of the overall study population

Find-AF 2 recruited a typical ischaemic stroke population. The mean age of 72 years is rather high compared to other recent stroke trials like CRYSTAL AF [5], PER DIEM [7], STROKE AF [8] and MonDAFIS [9] as well as all ESUS trials [14–16], which can easily be explained by the age cut-off of ≥ 60 years in Find-AF 2. The high proportion of patients aged 75 years and above is valuable for translation of study findings in clinical practice, since older patients are often underrepresented in clinical trials, while this group probably holds a high risk for AF. The overall Find-AF 2 trial population is nearly identical to the forerunner Find-AF_randomised_ trial [6] and comparable to previous heart rhythm monitoring and ESUS study populations in terms of the proportion of women, cardiovascular risk factors and a rather low NIHSS at admission [4–9, 14–16].

The most obvious difference results from the inclusion and exclusion criteria, which allowed the recruitment of patients with different stroke aetiologies in Find-AF 2, while in CRYSTAL-AF [5] and EMBRACE [4] only patients with cryptogenic stroke and in STROKE-AF [8] only patients with large or small vessel atherosclerosis were included. We believe that this approach is justified for the following three reasons: (1) AF and atherosclerosis share the same cardiovascular risk factors [29, 30],(2) previous studies have shown that the AF detection rate is comparable over different index stroke aetiologies [6, 8, 9], and(3) the aetiology of recurrent strokes often differs from that of the index stroke [31], suggesting that screening for AF is useful regardless of the suspected aetiology of the index stroke. Additionally, the Find-AF 2 trial will allow for assessment the effectiveness in different suspected stroke aetiologies.

Importantly, the vast majority of patients in Find-AF 2 were randomised within the first week after the index stroke, whereas the mean interval between stroke and randomisation was longer in most other heart rhythm monitoring studies [4, 5, 7]. With the exception of the ATTICUS trial, patients in the ESUS studies [14–16], patients were randomised weeks to months after index stroke which hampers translation to acute stroke care, whereas the results of the Find-AF 2 trial can easily be applied to patients treated on stroke units.

Differences in trial populations may influence the detection rate of AF as well as the number of recurrent strokes within the trials. The prevalence of AF, for example, is higher in elderly stroke patients, which makes prolonged Holter ECG highly efficient in this group of patients [32]. Excluding individuals younger than 60 years of age may therefore increase AF detection rate in Find-AF 2 and age distribution might explain lower rates of detected AF within other cohorts [9].

Furthermore, the incidence of recurrent strokes is particularly high within the first days after the index stroke [33]. If patients are included more than a month later as in some of the aforementioned trials [4, 5, 7, 14, 15, 17] the rate of stroke recurrence will probably be lower and some episodes of AF may be missed if rhythm monitoring begins weeks after stroke.

### Baseline characteristics by high- and low-risk strata

ESVEA was detected in the 24-h Holter ECGs in 22% of the Find-AF 2 trial population. This is in line with previous stroke populations [22, 24]. Despite different definitions for ESVEA, former trials agree that ESVEA is a predictor of AF detection in TIA or ischaemic stroke patients [21, 22, 34].

Patients in the high-risk stratum in our trial were significantly older and were less likely to be smokers. The index stroke was more severe, more frequently non-lacunar and cryptogenic. Information about comorbidities and baseline characteristics of ischaemic stroke patients with ESVEA is sparse with respect to the currently available studies. In the MonDAFIS trial patients with ESVEA were reported to be older as well as to have a higher median CHA_2_DS_2_-VASc score and baseline NIHSS score than patients without ESVEA or AF in an ECG monitoring during the acute phase of ischaemic stroke or TIA [22]. In our Find-AF 2 cohort, we found higher CHADS_2_ scores in ESVEA patients did not persist after adjusting for age and gender.

### Anticoagulation based on AF detection versus ESUS

Individualised precision medicine customizes treatment for the individual patient. We consider Find-AF 2 a precision medicine trial because detection of AF by the individually risk-adapted study intervention (rhythm monitoring) entails treatment with guideline conform OAC.

Hence, the Find-AF 2 trial differs from ESUS trials in an important aspect: In Find-AF 2, OAC is triggered by the diagnosis of AF. AF suggestive markers were used for OAC trigger in the ESUS trials (e.g. brain imaging of large cerebral lesions suggestive for thrombus of cardiac origin, elevated natriuretic peptides, enlarged left atrium, etc.) and are used in the Find-AF 2 trial only for tailoring the study intervention by ESVEA-based risk stratification with consecutive individualization of the heart rhythm monitoring scheme. The Find-AF 2 approach requires firstly patients with detected AF are switched from antiplatelets to OAC and secondly that OAC reduces the risk of ischaemic stroke in these patients. The OAC rate in newly detected AF patients of Find-AF 2 cannot be evaluated at this stage, but data from the forerunner trial Find-AF_randomised_ suggests that OAC can be expected in 90% of patients with detected AF. Regarding efficacy of OAC for stroke prevention in patients with ICM detected subclinical AF, there is evidence from a subgroup analysis of the ARTESiA trial [35] in patients with previous TIA or stroke [36] that apixaban led to lower rates of recurrent strokes, whereas in NOAH-AFNET 6 Edoxaban failed to reduce rates of stroke, systemic embolism, and cardiovascular death [37]. Therefore, it remains unclear up to date whether every oral anticoagulant reduces ischaemic strokes in patients with ICM detected subclinical AF and the results of both subgroup analyses are not sufficient for broadly recommending OAC in stroke or TIA patients with subclinical ICM detected AF. [38]

Another advantage of FIND-AF 2 compared to the ESUS concept is that Find-AF 2 includes a broad variety of stroke aetiologies, whereas ESUS trials only represent one stroke subgroup neglecting a possible benefit of AF detection in non-ESUS-patients due to switch of recurrent stroke aetiologies and shared risk factors between stroke and AF. The baseline characteristics presented here show that only about half of patients in Find-AF 2 were classified as ESUS implying that the objective of including various stroke aetiologies (e.g. lacunar strokes) was met.

### Limitations

The risk-adapted scaling of AF screening intensity has not been used in other trials and is not recommended for this purpose in current guidelines, but ESVEA has proved to be one of the best predictors of AF development in stroke patients [21, 24, 34]. However, the risk-adapted approach of Find-AF 2 has limitations. AF detected by 7-day Holter ECG may be more clinically relevant than AF detected by continuous ECG monitoring. This may be because the AF burden in these patients is different with potential implications for the absolute stroke risk, but also on the efficacy of OAC [39].

Furthermore, the stratification into high and low risk for developing AF will facilitate sub-group analyses, but a direct comparison of repeated 7-day Holter ECG and continuous monitoring will not be possible. Finally, by design, patients < 60 years of age and patients with a higher degree of disability (modified Rankin Scale (mRS) > 2 points) as well as patients with certain causes of stroke such as ACI stenosis requiring surgical or interventional treatment were excluded, which limits generalisability.

## Conclusions

The Find-AF 2 trial has successfully completed recruitment of a large acute ischaemic stroke population shortly after the index event and with a broad variety of suspected stroke aetiologies. Overall, 22% of the patients have to be considered to belin at high risk for AF. Those at high risk were older and had more severe index strokes, which were more often non-lacunar and of cryptogenic aetiology at hospital discharge. The results of the trial will contribute to future guidelines whether intensified cardiac rhythm monitoring using continuous ECG monitoring is justified in the entire population or the subgroup of patients with ESVEA.

## Supplementary Information

Additional file 1.

## Data Availability

Data will be made available on reasonable request.

## Abbreviations

AF: Atrial fibrillation
ARCADIA: Apixaban to prevent recurrence after cryptogenic stroke in patients with atrial cardiopathy (trial)
ARTESiA: Apixaban for stroke prevention in subclinical atrial fibrillation trial
ATTICUS: Apixaban for the treatment of embolic stroke of undetermined source trial
BMI: Body mass index
CHADS_2_: Congestive heart failure, arterial hypertension, diabetes mellitus, history of stroke/transient ischaemic attack (doubled)
CHA_2_DS_2_-VA: Congestive heart failure, arterial hypertension, age ≥ 75 years (doubled), diabetes mellitus, history of stroke/transient ischaemic attack (doubled), vascular disease, age 65 to 74 years
CHA_2_DS_2_-VASc: Congestive heart failure, arterial hypertension, age ≥ 75 years (doubled), diabetes mellitus, history of stroke/transient ischaemic attack (doubled), vascular disease, age 65–74 years and sex category (female)
CE: Cardioembolic stroke (according to TOAST classification)
CONSORT: Consolidated Statement of Reporting Trials
CRYSTAL AF: Cryptogenic stroke and underlying atrial fibrillation trial
CT: Computed tomography
ECG: Electrocardiogram
EMBRACE: 30-Day cardiac event monitor belt for recording atrial fibrillation after a cerebral ischaemic event trial
ESVEA: Excessive supraventricular ectopic activity
i.a.: Intraarterial
ICM: Implantable cardiac monitor
i.v.: Intravenous
LAA: Stroke due to large-artery atherosclerosis (according to TOAST classification)
MonDAFIS: Systematic monitoring for detection of atrial fibrillation in patients with acute ischaemic stroke trial
NAVIGATE ESUS: New approach rivaroxaban inhibition of Factor Xa in a global trial versus acetylsalicylic acid to prevent embolism in Embolic Stroke of Undetermined Source trial
NIHSS: National Institute of Health Stroke Scale
NOAH-AFNET 6: Non–Vitamin K Antagonist Oral Anticoagulants in Patients with Atrial High Rate Episodes trial
OAC: Oral anticoagulation
OC: Stroke due to other cause (according to TOAST classification)
PER DIEM: Post-embolic rhythm detection with implantable versus external monitoring trial
PROBE: Prospective randomised open-label multicentre trial with blinded endpoint assessment
RE-SPECT ESUS: Randomised, double-blind, evaluation in secondary stroke prevention comparing the efficacy and safety of the oral thrombin inhibitor dabigatran etexilate versus acetylsalicylic acid in patients with Embolic Stroke of Undetermined Source trial
STOKESTOP: Systematic ECG screening for atrial fibrillation among 75 year old subjects in the region of Stockholm and Halland, Sweden trial
SVO: Stroke due to small vessel occlusion (according to TOAST classification)
TIA: Transient ischaemic attack
TOAST: Trial of ORG 10172 in Acute Stroke Treatment
UC: Stroke due to unknown cause/cryptogenic stroke (according to TOAST classification)
