# Supplementary material for: Baseline characteristics of patients with acute ischaemic stroke included in the randomised controlled Find-AF 2 trial
Source: Neurol Res Pract. 2025 Jun 26;7(1):45. doi: 10.1186/s42466-025-00399-8 (PMC12203715; doi:10.1186/s42466-025-00399-8)
Supplement: Supplementary file 1 — Additional file 1. [file 42466_2025_399_MOESM1_ESM.docx]

|  | All patients  (n = 5227) | Control  (n = 2609) | Intervention  (n = 2618) | Low risk for AF  (n = 4075) | High risk for AF  (n = 1152) |
| --- | --- | --- | --- | --- | --- |
| Sex | 0 | 0 | 0 | 0 | 0 |
| Age | 0 | 0 | 0 | 0 | 0 |
| BMI | 134 | 62 | 72 | 100 | 34 |
| Smoking | 135 | 66 | 69 | 96 | 39 |
| Alcohol consumption | 203 | 92 | 111 | 156 | 47 |
| Previous stroke | 49 | 20 | 29 | 39 | 10 |
| Previous TIA | 48 | 20 | 28 | 37 | 11 |
| Systematic embolism | 21 | 10 | 11 | 13 | 8 |
| Heavy bleeding | 15 | 8 | 7 | 10 | 5 |
| Coronary artery disease | 29 | 15 | 14 | 19 | 10 |
| Myocardial infarction | 24 | 13 | 11 | 16 | 8 |
| Heart failure | 24 | 13 | 11 | 16 | 8 |
| Peripheral artery disease | 21 | 12 | 9 | 16 | 5 |
| Currently requires dialysis | 16 | 8 | 8 | 10 | 6 |
| Diabetes mellitus | 16 | 10 | 6 | 10 | 6 |
| Arterial hypertension | 4 | 1 | 3 | 3 | 1 |
| Dyslipidaemia | 23 | 11 | 12 | 18 | 5 |
| High risk for AF | 0 | 0 | 0 | 0 | 0 |
| Time from index stroke to randomisation | 1 | 0 | 1 | 1 | 0 |
| Type of index stroke | 0 | 0 | 0 | 0 | 0 |
| TOAST classification of index stroke | 38 | 17 | 21 | 34 | 4 |
| ESUS | 0 | 0 | 0 | 0 | 0 |
| i.v. or i.a. thrombolysis | 5 | 1 | 4 | 5 | 0 |
| NIH Stroke Scale at admission | 0 | 0 | 0 | 0 | 0 |
| CHA_2_DS_2_ score prior to stroke | 4 | 0 | 4 | 4 | 0 |
| CHA_2_DS_2_-VA score prior to stroke | 4 | 0 | 4 | 4 | 0 |
